# Determine and compare limb specific predictors based on the foot pressure index of right and left foot among young adults in Saudi Arabia using machine learning models

**DOI:** 10.1371/journal.pone.0360520

**Published:** 2026-10-23

**Authors:** Paul Silvian Samuel, Resmi Ann Thomas, Venkata Nagaraj Kakaraparthi, Ravi Shankar Reddy, Mastour Saeed Alshahrani, Hani H. Alnakhli, Batool Abdulelah Alkhamis, Kumar Gular

**Affiliations:** 1 Department of Medical Rehabilitation Sciences, College of Applied Medical Sciences, King Khalid University, Abha, Saudi Arabia; 2 College of Business, Centennial College, Toronto, Canada; Texas State University, UNITED STATES OF AMERICA

## Abstract

Foot posture is a key determinant of limb-biomechanics, gait balance as well as functional mobility. Usage of foot pressure index (FPI) is widespread to but the determinants of FPI without accounting to the left right foot differences. Therefore, identifying limb-specific determinants would enhance screening accuracy and help in more targeted interventions. The objective of the study was to determine and compare limb specific predictors based on the foot pressure index of right and left foot among young adults using machine learning models. A total of 581 (290 females and 291 males) 18–25-year-old young adults were assessed on anthropometric measures, joint hyper mobility and functional mobility. Using SAS-Viya, separate entropy-based decision trees, along with logistic regression models were developed and model performance was evaluated using validation-test accuracy and area under the curve (AUC). Decision tree models outperformed logistic regression for both FPI-Left (FPI-L) and FPI-Right (FPIR). FPIR was primarily predicted by joint hyper mobility, followed by stair ascent time, stability as well as BMI with stronger influence of both structural and load related factors. FPIL was predicted by closed-eye balance along BMI, open eye balance, stair ascent, Beighton score, underscoring the role of sensory motor control. The findings of the study reflect that young adults foot posture is complex, limb specific interactions among anthropometric, functional and sensory factors.

## Introduction

Assessment of foot posture is considered crucial in clinical practices checking musculoskeletal imbalances, rehabilitation as well as in prediction of future injuries. For this reason, the foot posture index, which is a widely established, reliable, multi-dimensional method is used to quantify foot alignment, by categorizing postures from highly supinated to highly pronated [1–3]. Analyzing plantar pressures during gait is very crucial for understanding foot function, biomechanics and has many implications on various activities of daily living as well as clinical conditions [4].

Recent evidence suggests that foot posture also influences lower-limb kinematics, dynamic balance shock absorption and load transmission throughout the kinetic chain. Studies have also reported that pronated and supinated foot types demonstrate plantar pressure distributions that are distinct, as well as show movement functionalities.

Foot posture plays a central role in both injury prevention as well as running performance and among them foot posture index has gained widespread acceptance because it is simple, cost effective and is able to classify foot posture into pronated, supinated as well as normal categories [5]. Recent research has used preoperative clinical phenotype approach with patients of end stage knee osteoarthritis identifying gait and pain [6]. Such gait and phenotype are particularly relevant to foot pressure index assessment due to its influence on plantar load distribution, and integrating FPI into phenotypic stratification may support targeted physiotherapy interventions and improved postoperative outcomes [7].

The FPI- 6 has shown to have high correlation between foot kinematics indicating that foot pressure index provides more complete description of foot posture [8] (Redmund et.al., 2006). It is important to understand that deviations in foot pressure most often known as supination and pronation, can lead to redistribution of foot loads thereby leading to future musculoskeletal problems [9,10].

Anthropometric variables like height, weight and foot size are found to explain almost 7.7% of FPI variation in 150 asymptomatic children [11]. Another study found significant relation with age and pathology, but interestingly not with gender or BMI, when studied on more than 1648 normative observations [12]. A 2012, research conducted on FPI total score, found that foot size, height and BMI explained 10.1% of overall foot pressure [13]. Foot pressure index on the right foot is found to be a predictive factor for foot pain especially among young women with higher pronation and BMI using multivariate logistic regression [14].

In another study to understand diabetes [15], Ahroni et al., 1999, found that BMI/weight was the only variable that remained as a significant predictor of a backward elimination logistic regression examining high pressure sites of foot, out of BMI, sex, age, race, as well as insulin use. Age, gender, weight and height were match controlled in subjects without diabetes, and found that BMI, gender and height were significantly related to foot pressure [16].

Beyond anthropometric characteristics, joint hyper mobility has acted as an important factor that influences foot posture and lower limb biomechanics. Due to increased ligamentous laxity, individuals with generalized joint hyper mobility demonstrates increased foot pronation, altered plantar loading as well as reduced postural stability. Such findings suggests that structural factors could interact with body compositions as well as functional performance measures in determining foot posture characteristics.

Balance conditions are also found to be significant in predicting foot pressure measure, especially for sway velocity under closed eye conditions, but failed to show relationship with open eye postural sway [17]. For peak pressures of right forefoot in a study conducted among young healthy adults with eyes open and closed, specific trends were recognized. With closed eyes the body movement was more that the participants exhibited a significantly greater length–surface function and a reduced center-of-pressure (COP) sway area, accompanied by lower mean and peak plantar pressures at the right forefoot [18]. Recent investigations have further demonstrated that visual deprivation, proprioceptive deficits and impaired postural control have been associated with compensatory changes in loading patterns.

When walking down and upstairs, adults both elderly and young showed greater side to side body leaning. However, interestingly, younger adults were able to reduce this leaning during descent compared to older adults [19]. Although foot postures are commonly assessed bilaterally, evidence suggests that such asymmetries can occur due to limb dominance, neuromuscular control and variations in loading behaviour. Furthermore, many previous studies have relied on conventional statistical approaches such as correlation and regression analysis. While these methods are useful for identifying associations, they may not adequately capture the complex nonlinear associations that exists between anthropometric, biomechanical, sensory and functional variables. Machine learning models, especially decision tree models, provide alternative approach capable of identifying hierarchical predictor relationships and clinically interpretable threshold values.

It is important to see that some or more of such studies have not attempted to study the predictors and splits of right foot and left foot differently. This research is therefore attempting to predict right and left foot posture index using decision trees, to identify and manage right-left pressure indexes specifically to enable rapid screening and early preventive intervention, identifying limb-specific determinants would enhance screening accuracy and help in more targeted interventions.

The objective of the study was to determine and compare limb specific predictors based on the foot pressure index of right and left foot among young adults using machine learning models.

## Materials and methods

### Study design and participant recruitment

This investigation was designed as a cross-sectional descriptive analytical study. A total of 581 asymptomatic young adults (291 men and 290 women) were recruited from the Physiotherapy Outpatient Department at King Khalid University, Saudi Arabia. Sample size adequacy was determined in advance using statistical power analysis conducted with G*Power software (version 3.1.9.4; Universität Kiel, Germany).The source population comprised 1,162 eligible individuals identified from university student and faculty registries. A systematic sampling approach was implemented, whereby the sampling interval was calculated by dividing the total eligible population by the desired sample size. Based on this interval, every alternate eligible individual was selected sequentially until the required sample size was achieved.

University includes more than 170 academic departments. Four colleges were randomly selected for inclusion. Recruitment was carried out within these colleges following formal administrative approval. Prior to participation, all individuals were provided with detailed information regarding the objectives and procedures of the study. Written informed consent was obtained from each participant. The participants recruitment and the study was conducted during 12/12/2024 till 18/11/2025. Ethical approval was granted by the King Khalid University, Institutional Ethical Committee (ECM #2019−60).

### Eligibility criteria

Participants aged between 18–25 years were considered eligible if they were free from current or recent foot-related complaints, demonstrated normal structural foot alignment, and exhibited intact sensory function without signs of peripheral neuropathy. Individuals with a history of lower-extremity orthotic use were excluded. (Fig 1)

**Fig 1:**
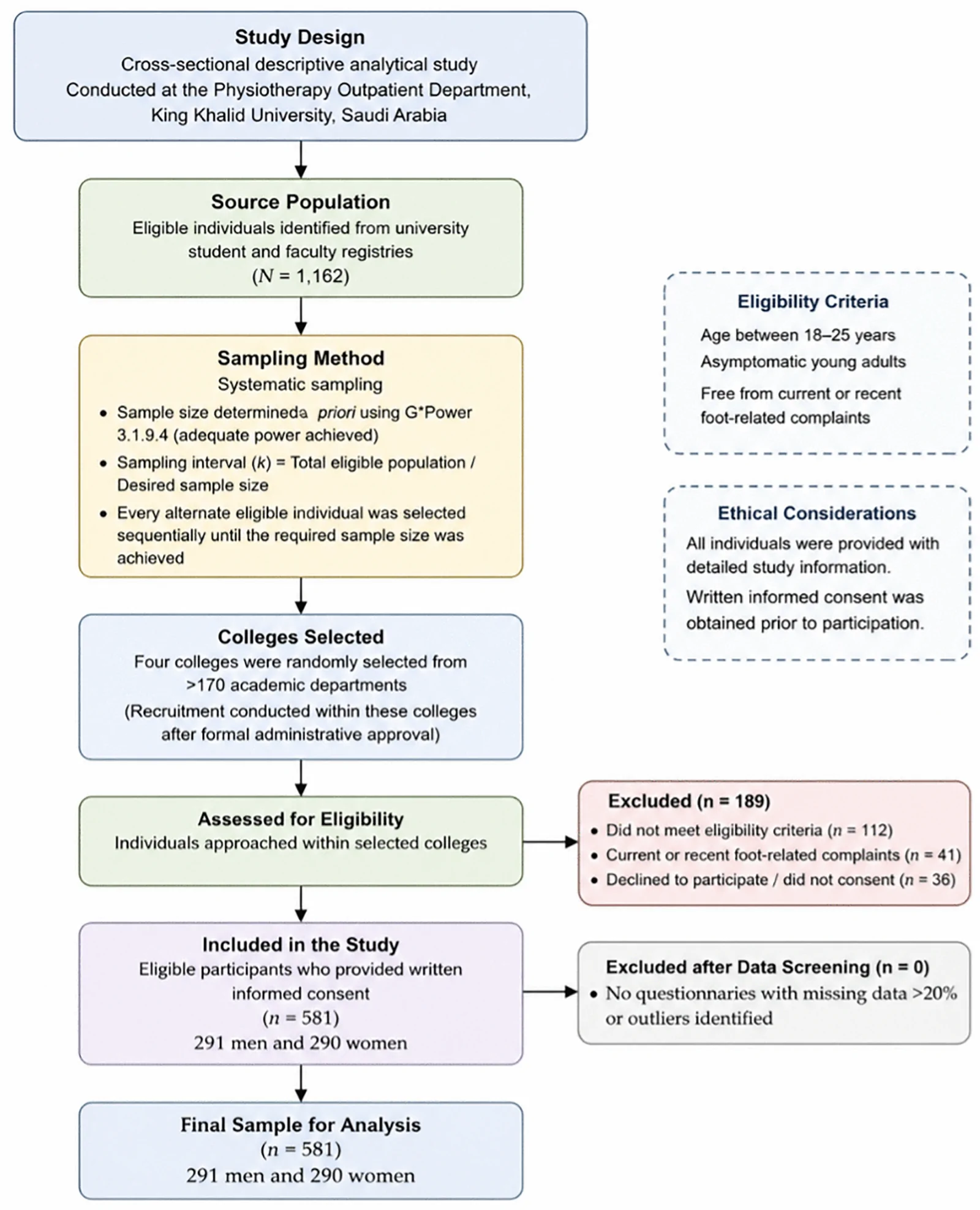
Flow Chart. Study Design and Participant Recruitment.

Exclusion criteria included any diagnosed musculoskeletal disorder, previous spinal or lower-limb surgery, congenital deformities, vestibular impairments, limb-length discrepancy, plantar heel pain, systemic diseases affecting balance, foot pain within the preceding six months, painful dermatological foot conditions, or pregnancy.

### Data collection procedures

Demographic variables including age and sex were recorded at baseline. Anthropometric parameters such as height, body weight, and body mass index. The BMI was measured using standardized procedures with a calibrated scale and stadiometer. Participants were measured without shoes and in light clothing, with weight recorded to the nearest 0.1 kg and height to the nearest 0.1 cm, after which BMI was calculated as weight (kg) divided by height squared (m^2^).

Participants then underwent a structured assessment protocol evaluating foot posture, static balance, stair negotiation performance, and generalized joint mobility. All evaluations were conducted by a single clinician with more than 16 years of professional experience to minimize inter-examiner variability.

### Assessment of foot posture

Foot alignment was assessed using the Foot Posture Index (FPI). Participants stood barefoot in a relaxed stance after briefly marching in place to assume their natural posture. Each foot was evaluated across six criteria: talar head palpation, curvature above and below the malleoli, prominence of the talonavicular joint, calcaneal alignment in the frontal plane, forefoot abduction/adduction, and medial longitudinal arch height.

Each criterion was scored on a five-point scale ranging from −2 to +2. The sum of these scores determined foot posture classification: highly supinated (−12 to −5), supinated (−4 to −1), neutral (0 to +5), pronated (+6 to +9), or highly pronated (+10 to +12). All assessments were performed by the same examiner in accordance with established reliability standards. These values were later categorized to normal and abnormal, with FPIR categories at 294 normal and 287 abnormal and for FPIL normal categories at 288 and 293 abnormal.

### Evaluation of static balance

Postural stability was measured using a near-tandem stance test. Participants stood barefoot with one foot positioned in front of the other, maintaining approximately 2.5 cm of lateral separation, with the heel of the front foot placed 2.5 cm ahead of the great toe of the rear foot. With eyes closed, participants maintained this position until balance was lost. The duration of successful stance was recorded in seconds.

### Stair climbing performance

Functional stair performance was assessed using a timed stair ascent and descent task. Participants ascended and descended eight indoor steps barefoot at a brisk yet safe pace. Each step measured 15 cm in height and 27.5 cm in depth. A handrail was available if necessary for safety. Timing began when the first foot left the ground and ended upon completion of the task. Total performance time was documented in seconds.

### Assessment of generalized joint mobility

Generalized joint hypermobility was evaluated using the Beighton scoring system. The nine-point scale assesses bilateral flexibility of the fifth metacarpophalangeal joints, thumbs, elbows, knees, and trunk flexion. One point was assigned for each positive maneuver, yielding a maximum score of nine. A score of five or more was considered indicative of generalized joint hypermobility.

SAS- VIYA 4, was used to create machine learning model. Data was preprocessed using built in replacement (to ensure outliers are replaced under +_3 standard deviations) and data partitioned as training (60%), validation (50%) and test (10%), for model development and evaluation. Machine learning models were developed using decision trees and logistic regressions. Decision trees were run using CHAD and entropy, like wise logistic regression using forward, backward and stepwise as well as fast backward regression as competitors. To control overfitting, and to select the optimal tree, cost-cutting pruning that starts with a large tree and remove branches that have the lowest predictive values. Automatic subtree selection was used to identify the optimal tree structure based on validation performance. Model tuning and pruning decisions were supported through 10-fold cross validation. The final tree, therefore, was pruned at 10 terminal leaves. The targets since were mostly balanced, target variables were taken at 0.5 probability. Model performance was evaluated using accuracy, area under the curve as well as misclassification rate. Final model selection was based on validation performance using SAS VIYA’s model comparison framework and decision trees were examined to derive clinically interpretative results.

## Results and discussion

The anthropometric characteristics of the study sample indicated that participants were predominantly young adults aged between 18–26 years and overall height ranged from 1.41 m to 1.89 m, body weight from 35–130 kg and BMI from 14.36 to 44.70 kg/ m^2^. Foot posture for both sides ranged from – 7 to +12, while Beighton scores varied between 0–9. Functional performance demonstrated stair ascent between 1. 92 and 5.66 seconds, and descent from 1.50 to 4.78s. Balance ranged from 1.24 to 28.98s with eyes closed and 2.52 to 41.47 with eyes open and the study was conducted among 290 females and 291 males.

Normality was assessed using the Shapiro–Wilk test and homogeneity of variance using Levene's test. Independent-samples t-tests were used to compare continuous variables between groups. To quantify the magnitude of differences, Cohen's d effect sizes and their 95% confidence intervals were calculated and reported alongside p-values.

Univariate analysis using independent sample t-test, revealed significant differences between foot pressure index right (FPIR) and left (FPIL). These categories were analyzed for significant mean differences among variables like age, height, weight, BMI, stair ascent, descent, balance with eyes closed and open. Interestingly for FPIR, age, height and balance with eyes open showed significant difference between normal (FPI values of 0–5) and abnormal (FPI of pronated, highly pronated, supinated and highly supinated) foot pressures. Age showed slightly older adults (22.02 ± 1.33), shorter in height (1.56 ± 0.07) and better balance with eyes open (21.87 ± 8.34) for right foot. Left foot pressure index showed no statistically significant differences among anthropometric, functional or balance variables at p < 0.05 but balance with eyes closed showed a mean difference at p < 0.10 towards poorer performance. (Table 1)

**Table 1:** The anthropometric characteristics of the study sample and Univariate analysis using independent sample t-test, revealed significant differences between foot pressure index right (FPIR) and foot pressure index left (FPIL).

Variable	FPIR = 0 Mean ± SD	FPIR = 1 Mean ± SD	*p*	FPIL = 0 Mean ± SD	FPIL = 1 Mean ± SD	*p*
Age (years)	21.24 ± 1.34	22.02 ± 1.33	**0.001**	21.60 ± 1.49	21.39 ± 1.18	.176
Height (m)	1.58 ± 0.05	1.56 ± 0.07	**0.015**	1.57 ± 0.06	1.58 ± 0.06	.339
Weight (kg)	55.36 ± 9.73	56.25 ± 11.87	0.515	56.36 ± 10.70	54.54 ± 10.24	.150
BMI (kg/m²)	22.10 ± 3.52	23.06 ± 4.61	0.066	22.75 ± 3.69	21.94 ± 4.38	.107
Stair Ascent	3.09 ± 0.66	3.23 ± 0.81	0.127	3.15 ± 0.70	3.12 ± 0.75	.759
Stair Descent	2.68 ± 0.61	2.78 ± 0.63	0.197	2.71 ± 0.58	2.71 ± 0.68	.998
Balance Eyes Open	19.73 ± 8.24	21.87 ± 8.34	**0.036**	20.16 ± 8.92	21.12 ± 7.25	.321
Balance Eyes Closed	8.21 ± 5.33	9.07 ± 5.18	0.182	8.13 ± 5.38	9.19 ± 5.06	.092

Effect size analysis using Cohen's d demonstrated a moderate effect for age (d = −0.58, 95% CI: −0.83 to −0.34). Small effects were observed for height (d = 0.34, 95% CI: 0.09 to 0.58), BMI (d = −0.24, 95% CI: −0.48 to −0.00), and balance with eyes open (d = −0.26, 95% CI: −0.50 to −0.02). All other variables exhibited trivial effect sizes (|d| < 0.20), indicating limited practical significance. For the FPIL comparison, all effect sizes were trivial to small (|d| = 0.00–0.21). (Table 2)

**Table 2:** Effect size analysis using Cohen's d for age, Height, BMI, Stair accent and decent and balance with eyes open and closed differences between foot pressure index right (FPIR) and foot pressure index left (FPIL).

Variable	Mean±SD FPIR = 0	Mean±SD FPIR = 1	p-value	Cohens D	Mean±SD FPIL = 0	Mean±SD FPIL = 1	p-value	Cohens D
Age (years)	21.2 ± 1.34	22.0 ± 1.33	0.001	−0.5 (−0.8, −0.3)	21.6 ± 1.4	21.3 ± 1.1	0.17	0.1 (−0.08, 0.3)
Height (m)	1.5 ± 0.05	1.56 ± 0.07	0.01	0.3 (0.09, 0.58)	1.5 ± 0.06	1.5 ± 0.06	0.33	−0.1 (−0.3, 0.1)
Weight (kg)	55.3 ± 9.7	56.2 ± 11.8	0.51	−0.08 (−0.32, 0.16)	56.3 ± 10.7	54.5 ± 10.2	0.15	0.1 (−0.07, 0.4)
BMI (kg/m²)	22.1 ± 3.5	23.0 ± 4.6	0.06	−0.24 (−0.48, −0.00)	22.7 ± 3.6	21.9 ± 4.3	0.10	0.2 (−0.03, 0.4)
Stair Ascent	3.0 ± 0.6	3.2 ± 0.8	0.12	−0.2 (−0.4, 0.04)	3.1 ± 0.7	3.1 ± 0.7	0.75	0.04 (−0.2, 0.2)
Stair Descent	2.6 ± 0.6	2.7 ± 0.6	0.19	−0.1 (−0.40, 0.08)	2.7 ± 0.5	2.7 ± 0.6	0.99	0.00 (−0.2, 0.2)
Balance Eyes Open	19.7 ± 8.2	21.8 ± 8.3	0.03	−0.26 (−0.50, −0.02)	20.1 ± 8.9	21.1 ± 7.2	0.32	−0.1 (−0.3, 0.1)
Balance Eyes Closed	8.21 ± 5.33	9.07 ± 5.18	0.182	−0.16 (−0.40, 0.08)	8.13 ± 5.38	9.19 ± 5.06	0.092	−0.20 (−0.44, 0.04)

Two separate machine learning models were created to understand the predictors of right foot pressure index and left foot pressure index differently. Logistic regression models did not reach the cutoff criteria for accuracy or ROC area under the curve specification and decision trees were better describing the models to understand the predictors for both the models. Test results are taken for final comparison since Steyerberg et al., 2013 [20],emphasized that test data should be treated as a proxy for unforeseen patients, providing an estimate as to how the final model is expected to perform in real world clinical settings. (Fig 2)

**Fig 2:**
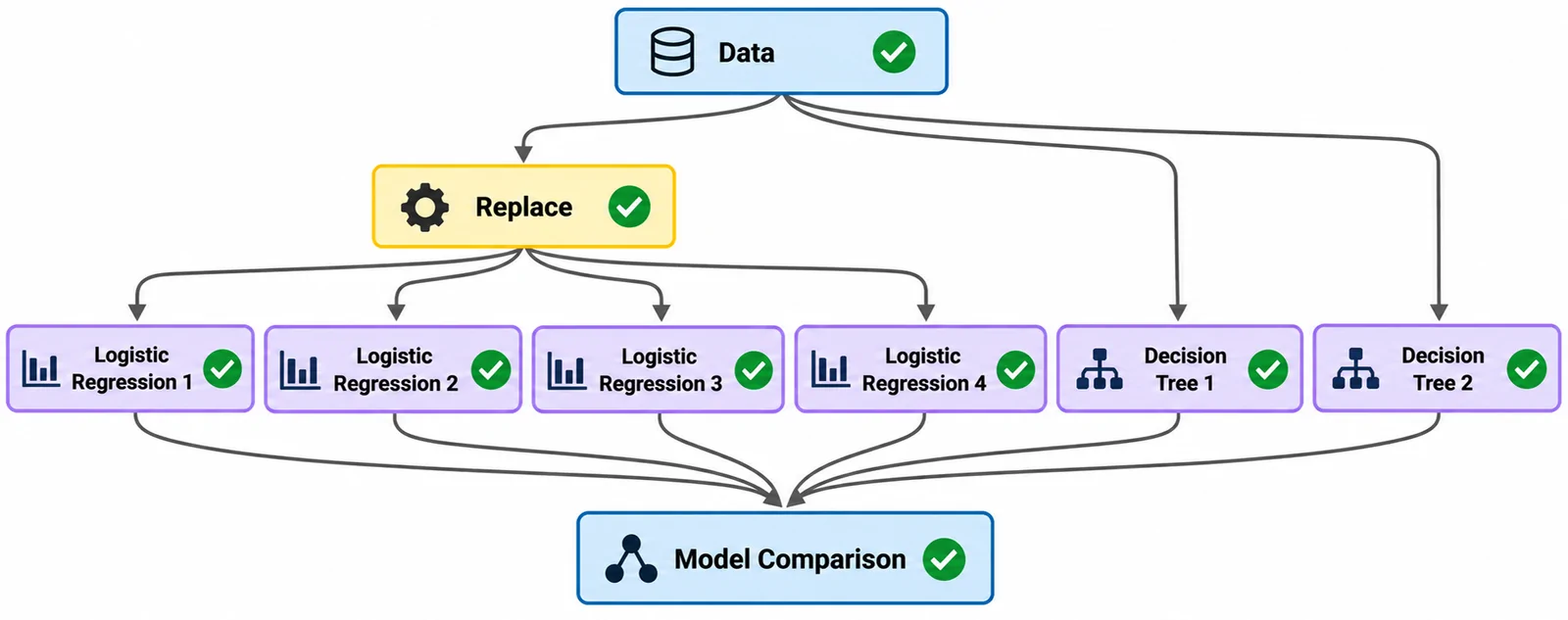
Decision Tree. Machine learning model pipeline.

The decision tree produced 10 terminal nodes and the primary split for Foot Pressure index for Right foot was Beighton scale with individuals scoring below 5 is classified as normal FPI. Among participants with Beighton scores ≥ 5, further stratification was determined by **stair ascent performance**, followed by **stature and body mass index**. Specifically, abnormal FPIR classification occurred in subgroups combining higher Beighton scores with reduced stair ascent performance and either shorter stature (< 1.47 m) or elevated BMI (≥ 43.92). Whereas, with higher Beighton scores but has a stair ascent performance (≥ 2.11 s) or greater were predominantly classified as normal. Residual branches indicated that, in a small subset, elevated Beighton scores alone were sufficient to predict abnormal FPIR, reflecting structural heterogeneity in right foot posture. (Table 3)

**Table 3:** Decision Tree Model Performance and Characteristics for Right and Left Foot Pressure Index Classification.

Characteristic	FPIR Foot Pressure Index- Right	FPIL Foot Pressure Index- Left
Target Variable	FPIR (Normal vs Abnormal)	FPIL (Normal vs Abnormal)
Final Model	Decision Tree (Entropy-based)	Decision Tree (Entropy-based)
Selection Criterion	Root Average Squared Error	Root Average Squared Error
Number of Terminal Leaves	10	10
Test data Accuracy	0.90	0.90
Misclassification Rate	0.10	0.10
Area Under ROC Curve (AUC)	0.89	0.97
Primary Split Variable	Beighton Scale	Body Mass Index (BMI)
Key Secondary Predictors	Age, Height, Weight, BMI	Balance (Eyes Open/Closed), Stair Ascent, Beighton Scale
Dominant Predictor Type	Hypermobility + Anthropometrics	Functional Balance + Mobility
Overall Model Assessment	Good predictive performance	Excellent predictive performance

For foot pressure index – Right, the final decision tree comprised of 11 terminal leaves and was taken based on the lowest average squared error and showed a test accuracy of 0.90 and area under the curve of 0.89, indicating good discriminatory performance. The primary model split was Beighton scale <25. 25 which predicted abnormality of the foot, followed by weight < 46, and then with <1.58 m of height with BMI of less than 43.92 predicts abnormality of right foot (supination, hyper supination, pronation and hyper pronation). (Table 4)

**Table 4:** FPIR (Right Foot Pressure Index) – Decision Tree.

Leaf ID	Full Logical Rule	Predicted FPIR
R1	Beighton Scale < 5	Normal
R2	Beighton Scale ≥ 5, Stair Ascent < 2.11, Height < 1.47	Abnormal
R3	Beighton Scale ≥ 5, Stair Ascent < 2.11, Height ≥ 1.47	Normal
R4	Beighton Scale ≥ 5, Stair Ascent ≥ 2.11	Normal
R5	Beighton Scale < 5, Stair Ascent < 2.11, Height < 1.47	Abnormal
R6	Beighton Scale < 5, Stair Ascent < 2.11, Height ≥ 1.47	Normal
R7	Beighton Scale ≥ 5, Stair Ascent < 2.11, BMI ≥ 43.92	Abnormal
R8	Beighton Scale ≥ 5, Stair Ascent < 2.11, BMI < 43.92	Normal
R9	Beighton Scale ≥ 5, Stair Ascent ≥ 2.11 (alternate branch)	Normal
R10	Beighton Scale ≥ 5 (residual terminal branch)	Abnormal

For left foot pressure index (FPIL), the entropy-based decision tree yielded ten terminal leaves and most of the leaves were dominated by balance-related variables. The primary split occurred at a balance with eyes closed, where people showed superior postural stability (3.81 s) was classified as normal. For people with poor balance (<3.81s), subsequent divisions were lower body mass index (<21.21), reduced visual dependent balance (eyes open <21.10 s) and lower Beighton scores (<14.80) and slower stair times (<31.76s). In contrast participants with higher BMI (>21.21), preserved balance, or higher Beighton thresholds (>21.94) were predominantly classified as normal. Residual and alternate branches largely was for normal classification, underscoring the strong influence of functional balance on left foot posture (Table 5).

**Table 5:** FPIL (Left Foot Pressure Index) – Entropy-Based Decision Tree.

Leaf ID	Full Logical Rule	Predicted FPIL
L1	Balance Eyes Closed < 3.81	Normal
L2	Balance Eyes Closed ≥ 3.81, BMI < 21.21, Balance Eyes Open < 21.10, Beighton < 14.80	Abnormal
L3	Balance Eyes Closed ≥ 3.81, BMI < 21.21, Balance Eyes Open < 21.10, Beighton ≥ 14.80	Normal
L4	Balance Eyes Closed ≥ 3.81, BMI < 21.21, Balance Eyes Open ≥ 21.10, Stair Ascent < 31.76	Abnormal
L5	Balance Eyes Closed ≥ 3.81, BMI < 21.21, Balance Eyes Open ≥ 21.10, Stair Ascent ≥ 31.76	Normal
L6	Balance Eyes Closed ≥ 3.81, BMI ≥ 21.21, Beighton < 21.94	Abnormal
L7	Balance Eyes Closed ≥ 3.81, BMI ≥ 21.21, Beighton ≥ 21.94	Normal
L8	Balance Eyes Closed ≥ 3.81, BMI < 21.21, Balance Eyes Open < 21.10 (alternate branch)	Normal
L9	Balance Eyes Closed ≥ 3.81, BMI ≥ 21.21 (alternate branch)	Normal
L10	Balance Eyes Closed ≥ 3.81 (residual terminal branch)	Normal

Taken together, the paired decision trees reveal a **clear functional asymmetry** between the left and right feet. The **left foot (**FPIL) appears to be predominantly influenced by **balance and postural control mechanisms**, particularly under conditions of reduced sensory input, whereas the **right foot (**FPIR) is more strongly shaped by **joint structure, mobility, and anthropometric factors**. This asymmetry suggests that the two feet may play **distinct functional roles** in maintaining posture and movement, with the left foot contributing more to **stability** and the right foot contributing more to **propulsion and load transmission.** Importantly, the split thresholds identified in these trees represent **algorithm-selected decision boundaries** and should not be interpreted as clinical diagnostic cut-offs. Instead, the models provide insight into the **hierarchical relationships among balance, mobility, hypermobility, and anthropometric factors** that underlie foot pressure distribution patterns.

Previous researchers have tried to examine the relationships between plantar pressure distribution, balance performance, anthropometric characteristics and functional mobility and have used predictive methods like decision trees in their studies on applied rehabilitation and biomechanical research [21,22]. The role of decision trees in identifying threshold-based rules linking plantar pressure variables with deficits in balance as well as functional impairment especially during challenging mobility tasks have also been demonstrated [19,23]. Likewise, Putti et al., 2007 [24] have proved the role of anthropometric variables such as body mass index and weight have frequently emerged as primary split variables, with stair performance as well as balance using decision trees.

The present study adds to current understanding of foot posture and plantar pressure behaviour in young adults by combining traditional biomedical assessment with machine learning interpretations. This integrated approach provides a more holistic framework identifying factors associated with abnormal foot pressure patterns. By incorporating anthropometric characters, functional mobility, joint hypermobility, balance into entropy-based decision trees, the analysis moves beyond simple associations and offers data driven clinically meaningful interpretation of pressure behaviour. The present study has reinforced previous research by showing significant relationship with anthropometric variables, balance, functional mobility, hyper mobility, for example height, body mass and index and weight affect foot structure and foot loading changes in skeletal alignment and stress transmission across medial longitudinal arch [25–28].

Increased adiposity can change how foot handles load by thickening plantar tissues, reduce arch stiffness which alters pressure patterns even among young normal population, without seeming musculoskeletal issues [27,29,30]. Such patterns match broader evidence showing that body weight and BMI lead to greater fore foot and mid foot abnormalities, altered gait mechanics and increased musculoskeletal loading [31,32].

The result of this study shows that dominant limb is responsible for propulsion and force generation while non dominant limb plays significant stabilizing role during stance as well as postural control [33,34]. These differences in functionality appear in distinct muscle activation strategies, joint profiles, patterns of load transfer which produces side specific plantar pressure behaviours [35].

Interestingly the structures of the FPIR and FPIL decision trees reflects how the two limbs are different when it comes to regulating plantar loadings. While the FPIR (right foot pressure index) is dominated by structural integrity and mechanical load transmission related variables, FPIL (left foot) shows predominance of balance performance as well as sensory motor integration, which is concurrent with existing literature.

Carpes et al., 2010 [34] has found that dominance of hypermobility as the first split of the right foot pressure is consistent with the fact that dominant foot is more engaged in propulsion and load acceptance during dynamic tasks. The role of Beighton scale, which quantifies generalized hyper mobility is found to reduce ligamentous restraint and joint stiffness compromising the foots ability to resist deformation under load [36]. The secondary role of stair ascent performance in the FPIR decision tree further elaborates the functional demands placed on the dominant limb, since stair ascent requires high concentric force, rapid weight transfer which are again easier associated with dominant limb [37]. It is interesting to note that anthropometric measures like height and extreme BMI appear as tertiary splits in the FPIR model, consistent with evidence that mechanical load magnitude and plantar contact metrics is critical for functional and structural buffers are compensated as noted earlier [26].

In contrast the FPIL decision tree is dominated by balance related variables where the primary determinant is balance with eyes closed, which aligns with previous research on non-dominant limb assuming stabilizing role contributing to postural control and sensory regulation [34]. Such eyes’ closed balance is reflective of the integrity of proprioceptive and plantar cutaneous feedback especially when how center of pressure movements and how weight is distributed across foot when vision is compromised.

The result of the study demonstrates that foot posture in young adults is governed by complex structural, functional, anthropometric and sensory factors that differs between both limbs. The use of decision tree modeling in this study provides clinically meaningful, threshold-based interruptions that enhance understanding of foot biomechanics and may inform targeted assessments and intervention strategies in rehabilitation and preventive care. The findings of this study have direct clinical relevance for assessment and intervention planning in young adults. The limb-specific predictors identified by the decision-tree models highlight the importance of bilateral foot assessment without assuming symmetry, as the dominant and non-dominant limbs may exhibit abnormal plantar pressure patterns for different underlying reasons.

Clinicians should consider incorporating balance testing under sensory-challenged conditions, functional mobility tasks such as stair ascent, and screening for joint hypermobility and elevated BMI alongside traditional foot posture assessment. The threshold-based rules generated by the decision trees offer a practical framework for early identification of individuals at risk of abnormal plantar loading, enabling targeted interventions such as balance training, load management strategies, weight management, or foot-specific strengthening to be tailored according to limb function rather than applying uniform bilateral approaches. The cross-sectional design of the study is a limitation to causal inference and even though decision tree helps in strong clinical interpretability, it’s a sample dependent analysis, which must be considered as a limitation to the research.

## Conclusions

The findings of the study have direct clinical relevance for assessment and intervention planning for young adults. The limb-specific predictors identified by the decision-tree models highlight the importance of bilateral foot assessment without assuming symmetry, as the dominant and non-dominant limbs are found to exhibit abnormal plantar pressure patterns for different underlying reasons. Clinicians should consider incorporating balance testing under sensory-challenged conditions, functional mobility tasks such as stair ascent, and screening for joint hypermobility and elevated BMI alongside traditional foot posture assessment. The threshold-based rules generated by the decision trees offer a practical framework for early identification of individuals at risk of abnormal plantar loading, enabling targeted interventions such as balance training, load management strategies, weight management, or foot-specific strengthening to be tailored according to limb function rather than applying uniform bilateral approaches.

However, the findings of the study should be interpreted based on the methodological constraint, since the research has used cross-sectional study design, which limits the ability to establish causal relationships. While the entropy-based decision tree identifies variables that improve classification accuracy, certain times these relationships can be influenced by other confounding variables as well. Further longitudinal and interventional studies are required to determine temporal and causal pathways underlying these associations. Future research should aim to validate these findings in larger and more diverse populations including children, older adults as well as athletes.

Overall, the study has successfully demonstrated that foot postures in young adults is influenced by complex and limb specific interactions among anthropometric, functional and sensory factors, supporting the need for individualized and evidence-based assessment strategies.

## Supporting information

S1 File S1 data. (XLSX)
